# Prenatal therapy in developmental disorders: drug targeting via intra-amniotic injection to treat X-linked hypohidrotic ectodermal dysplasia

**DOI:** 10.1186/1750-1172-9-S1-P10

**Published:** 2014-11-11

**Authors:** Holm Schneider, Pascal Schneider, Peter Krieg, AnhThu Dang, Kenneth Huttner, Katharina Hermes

**Affiliations:** 1 German Competence Centre for Children with Ectodermal Dysplasias, Department of Pediatrics, University Hospital Erlangen, 91054 Erlangen, Germany; 2 University of Lausanne, 1066 Epalinges, Switzerland; 3 German Cancer Research Center, 69120 Heidelberg, Germany; 4 Edimer Pharmaceuticals Inc., Cambridge, MA 02142, USA

## Background

Disorders that irremediably affect fetuses make early stage therapies desirable. X-linked hypohidrotic ectodermal dysplasia (XLHED), the most common inherited disorder of ectoderm development affecting the skin and its appendages, glands, and teeth, is caused by a lack of the signaling molecule ectodysplasin A1 (EDA1). In the *Tabby* XLHED mouse model, repeated intravenous administration of EDA1 to pregnant mice has been shown to correct the developmental abnormalities in the offspring. Maternal drug administration, however, exposes mothers to potential drug toxicity and is limited by the variability in transplacental drug delivery. Alternative approaches to fetal treatment should entail low risk drug delivery with reproducible pharmacokinetics. We hypothesized that a single injection of an EDA1 replacement molecule into the amniotic fluid could allow sustained drug exposure at levels sufficient for correction of XLHED.

## Materials and methods

A human IgG1:EDA1 fusion protein, EDI200, was tested for its stability in human amniotic fluid using a receptor-binding ELISA. It was injected into amniotic sacs of wild-type mice to evaluate fetal drug uptake and pharmacokinetics, and administered to *Tabby* mouse fetuses at doses between 1 and 100 µg/g fetal weight. Phenotypic correction was assessed.

## Results

EDI200 was demonstrated to be stable in amniotic fluid at 37°C for at least one week. Intra-amniotic administration of the highest dose resulted in substantial fetal uptake with mean serum levels of 9.0 and 1.2 µg/ml at 6 and 96 hours, respectively. Maternal serum levels remained <0.1 µg/ml. In *Tabby* mice, a single intra-amniotic EDI200 injection at day 15 of gestation restored normal ectoderm development in a dose-dependent manner. Doses of 10 μg/g fetal weight or above led to complete phenotypic correction of skin appendages, eccrine sweat glands, eye lids, and teeth. No adverse effects of the treatment were detected during an observation period of one year.

## Conclusions

Intra-amniotic protein application may lead to rapid and continuous fetal uptake, sustained serum levels, and therapeutic efficacy comparable with repeated maternal injection. It allows prenatal drug targeting with minimal maternal exposure and may, thus, represent a novel paradigm for treatment of disorders in early human development.

